# Editorial Notes

**Published:** 1893-04

**Authors:** 


### Editorial Notes.

DR. J. B. S. HOLMES, of Rome, Ga., called on us a few days ago.

DR. S. C. DREWRY, of Griffin, Ga., paid the RECORD a visit yesterday.

DR. D. W. WHITTAKER, the affable representative of Renz & Henry Manufacturing Pharmacists, of Louisville, Ky., dropped in to see us a few days ago.

WE desire to call the attention of the profession to the card of Dr. K. C. Divive, the Specialist in Rectal Diseases, which will be found in our advertising pages.

DR. Z. T. GRADY, of Lafayette, Ala., is in the city, having stopped over a few days en route home from New York, where he has been for the last three months taking a full course at the New York Post-Graduate School and Hospital.

DR. ALFRED F. HARRINGTON, of West Point, Ga., was in the city last week and favored us with a pleasant call. Dr. Harrington is one of the brightest young physicians in Georgia and has established a large and profitable practice in his home city and surrounding country.

The commencement exercises of the Fourth Annual Session of the Chattanooga Medical College was held in the opera house in that city on the evening of March 14th.

The graduating class numbered thirty, which is, indeed, an excellent showing for such a young institution.

Dr. C. L. Guice, the valedictorian. delivered an eloqueut address, which won the approbation of the vast audience.

Dr. G. A- Baxter addressed the class in behalf of the faculty, and his words of advice and expressions of good will, and the ties connecting the students to their Alma Mater, met with hearty applause from the class and audience.

Dr. Cobleigh and his associates are to be congratulated for their enterprise in establishing such a fine institution.

